# Bufadienolides of *Kalanchoe* species: an overview of chemical structure, biological activity and prospects for pharmacological use

**DOI:** 10.1007/s11101-017-9525-1

**Published:** 2017-08-02

**Authors:** Joanna Kolodziejczyk-Czepas, Anna Stochmal

**Affiliations:** 1 Department of General Biochemistry, Faculty of Biology and Environmental Protection, University of Lodz, Pomorska 141/143, 90-236 Lodz, Poland; 2 Department of Biochemistry, Institute of Soil Science and Plant Cultivation, State Research Institute, Czartoryskich 8, 24-100 Pulawy, Poland

**Keywords:** Bufadienolide, *Kalanchoe*, Cytotoxicity, Cancer therapy, Ethnomedicine

## Abstract

Toad venom is regarded as the main source of bufadienolides; however, synthesis of these substances takes also place in a variety of other animal and plant organisms, including ethnomedicinal plants of the *Kalanchoe* genus. Chemically, bufadienolides are a group of polyhydroxy C-24 steroids and their glycosides, containing a six-membered lactone (α-pyrone) ring at the C-17β position. From the pharmacological point of view, bufadienolides might be a promising group of steroid hormones with cardioactive properties and anticancer activity. Most of the literature concerns bufadienolides of animal origin; however, the medicinal use of these compounds remains limited by their narrow therapeutic index and the risk of development of cardiotoxic effects. On the other hand, plants such as *Kalanchoe* are also a source of bufadienolides. *Kalanchoe pinnata* (life plant, air plant, cathedral bells), *Kalanchoe daigremontiana* (mother of thousands) and other *Kalanchoe* species are valuable herbs in traditional medicine of Asia and Africa. The present review focuses on the available data on chemical structures of 31 compounds, biological properties and prospects for therapeutic use of bufadienolides from *Kalanchoe* species. Furthermore, it presents some new investigational trends in research on curative uses of these substances.

## Introduction

Bufadienolides are a group of polyhydroxy C-24 steroids and their glycosides. The first described bufadienolide was scillaren A, identified in Egyptian squill (*Scilla maritima*) (Stoll et al. 1933). The term “bufadienolides” originates from the genus *Bufo*—toads, which venom (a skin secretion) contains these compounds. Both animals (toads, snakes) and plants (Crassulaceae and Hyacinthaceae, in particular) synthesize bufadienolides, while the bufadienolide orthoesters were found only in several plant species: *Kalanchoe daigremontiana* Raym.-Hamet & H. Perrier, *Kalanchoe tubiflora* (Harv.) Raym.-Hamet, the hybrid *Kalanchoe daigremontiana* × *tubiflora*, *Kalanchoe pinnata* (Lam.) Pers., as well as in *Melianthus comosus* Vahl and *Bersama abyssinica* Fresen (Melianthaceae family).

The range of biological properties of bufadienolides includes cytotoxic, antitumor and cardiotonic activities (Gao et al. 2011), however, uncontrolled administration of these substances may induce the occurrence of side effects (Puschett et al. 2010). Most of the literature concerning the chemical characteristics, biological properties and possible therapeutic effects of bufadienolides includes data derived from studies on substances of animal origin. Chemistry and biological activities of bufadienolides synthesized by *Kalanchoe* plants are less known. Members of the *Kalanchoe* genus (Crassulaceae) are native for subtropical and tropical regions of Asia, Africa and America as well as for Australia and Madagascar. In Europe, *K. pinnata* and *K. daigremontiana* are mainly grown as house ornamental plants; however, their remedial properties are also known. Furthermore, both these and other *Kalanchoe* species are popular medicinal herbs in different regions of the world (Table 1). Traditional recommendations for using these plants include a wide range of diseases, including gastric ulcers, kidney stones, rheumatoid arthritis, bacterial and viral infections, skin diseases, cold as well as other disorders (e.g. Fürer et al. 2016; Kawade et al. 2014; Pattewar 2012; Rajsekhar et al. 2016). Ethnomedicinal uses of *Kalanchoe*-derived preparations are mostly based on internal or external administration of crude extracts or plant juice. There is no data on traditional uses of purified bufadienolides or semi-purified bufadienolide-rich preparations. However, available findings suggest that therapeutic activities (anti-cancer action, in particular) of *Kalanchoe*-derived medicines may be partly dependent on the presence of bufadienolides. Studies on these compounds, originated from various sources, revealed their anti-inflammatory, anti-cancer, anti-viral and other beneficial activities (Kamboj et al. 2013). Different research groups demonstrated anti-cancer properties of bufadienolides synthetized by *Kalanchoe* plants (e.g. Deng et al. 2014; Huang et al. 2013; Wu et al. 2006; Yamagishi et al. 1989). Daigremontianin and bersaldegenin-1,3,5-orthoacetate are listed in literature as sedative substances and natural adamantane derivatives (“trioxaadamantanes”) that may possess anti-influenza activity (Wanka et al. 2013). Additionally, the analysis of existing ethnomedicinal evidence (e.g. Botha 2016; Lans 2006; Süsskind et al. 2012), followed by studies with contemporary (bio)chemical and other scientific methods, may provide new data on safety or possible risk of using of *Kalanchoe* bufadienolide-containing extracts and preparations in humans.

**Table 1:** *Kalanchoe* species in ethnomedicine (a compilation of data)

Species (with English names)	Traditional uses and geographical region and/or country
*Kalanchoe crenata* (Andrews) Haw. (Never-die)	Medicinal plant, used during pregnancy by Anyi-Ndenye women (Eastern Ivory Coast, Africa) (Malan and Neuba 2011) Leaves are recommended to heal umbilical cord wounds in newborns (Mabira Central Forest Reserve, Uganda) (Tugume et al. 2016)
*Kalanchoe daigremontiana* Raym.-Hamet & H. Perrier, syn. *Bryophyllum daigremontianum* Raym.-Hamet & H. Perrier. (Mother of Thousands)	One of the most frequently prescribed anthroposophic medications, administered against psychic agitation, restlessness, and anxiety—studies conducted at Hospital Havelhoehe, Germany (Süsskind et al. 2012)
*Kalanchoe densiflora* Rolfe	For the treatment of wounds (Samburu of Mt. Nyiru, South Turkana, Kenya) (Bussmann 2006)
*Kalanchoe germanae* Raym.-Hamet ex Raadts (Air plant)	Removal of ganglion—the pound leaves are used on ganglion area (Kenya) (Kipkore et al. 2014)
*Kalanchoe glaucescens* Britten	Leaves are used to treat cough (Mabira Central Forest Reserve, Uganda) (Tugume et al. 2016)
*Kalanchoe gracilis* Hance, syn. *Kalanchoe ceratophylla* Haw.	To cure injuries, pain, fever and inflammation (Taiwan) (Lai et al. 2010)
*Kalanchoe laciniata* L. (Christmastree plant)	Juice from the leaves is used externally for joint pain (Southern India) (Karuppuswamy 2007) Powdered leaves are administered to alleviate cough, to cure colds and inflammation and for healing of boils and wounds (Southern India, Malaysia) Headache (Philippines) Crushed leaves are applied externally to decrease body temperature and to heal ulcers (Cambodia, Laos, Vietnam) To cure wounds, inflammation and diabetes (India) (Deb and Dash 2013)
*Kalanchoe lanceolata* (Forsk.) Pers.	Anti-malarial remedy (Kenya) (Njoroge and Bussmann 2006) The leaf juice is administered during dysentery (India) (Bapuji and Ratnam 2009)
*Kalanchoe marmorata* Bak.	Boiled juice is used as eye drops for treatment of eye infections (eastern Ethiopia) (Belayneh and Bussa 2014)
*Kalanchoe petitiana* A. Rich.	Leaf juice is applied on the fractured for bone setting (Ethiopia) (Ragunathan and Abay 2009)
*Kalanchoe pinnata* (Lam.) Pers., syn. *Bryophyllum pinnatum* Lam., *Bryophyllum calycinum* Salisb. (Life plant, air plant, love plant, Canterbury bells, Cathedral bells)	In the treatment of urinary bladder stones (India, Trinidad and Tobago) (Lans 2006; Sen et al. 2008) Leaf extract is used to cure amoebic dysentery (North Bengal) (Mitra and Mukherjee 2010) Wounds, bruises, swellings and insect bite (Himalaya) (Hussain and Hore 2007) Diarrhea (India) (Dash and Padhy 2006) Antibacterial and anti-inflammatory remedy (Vietnam) (Nguyen et al. 2004) Internally: to cure acute and chronic bronchitis, pneumonia and others respiratory tract infections, fever; externally: to treat dermatomycosis (Nigieria) (Okwu and Nnamdi 2011) Leaves are recommended for treatment of cough in adults and children (Kibale National Park, Uganda) (Namukobe et al. 2011) Inflammation, dermatosis, skin problems, wound healing, arthritis, asthma, bruises, diabetes, infections, tumours and ulcers—worlwide (Quazi Majaz et al. 2011a, b) Paste from macerated leaves is used externally for muscle and joint pain (Bangaldesh) (Tumpa et al. 2014) Preparations from leaves are used to treat digestive disorders (India) (Barukial and Sarmah 2011) Decoction from leaves is administered to remove kidney stones (Bangladesh) (Afroz et al. 2013) Leaves are chewed with salt as a remedy for dissolving of gall bladder stones (Bangladesh) (Rahmatullah et al. 2011) Herbal preparation from roots and leaves is administered to women for recovering after childbirth (West Java) (Sihotang 2011) Leaf paste is applied externally to treat scorpion bite (India) (Vaidyanathan et al. 2013) Leaf juice is recommended to treat cholera, diarrhea and dysentery (Bangladesh) (Khan et al. 2015) Leaves are used to treat urinary problems, incl. kidney and gall bladder stones (Bangladesh) (Bhowmik et al. 2014) Raw laves are chewed with sugar to treat dysentery and diarrhea; leaf juice is recommended to cure jaundice; leaf paste is used externally to heal skin infections and pimples (Bangladesh) (Das and Choudhury 2012)
*Kalanchoe tubiflora* Raym.-Hamet, syn. *Bryophyllum delagoense* (Eckl. & Zeyh.) Druce (Chandelier plant)	One of the most common medicinal plants used for wound healing (Brazil) (Hsieh et al. 2012)

This work reviews the available data on *Kalanchoe* species as a source of bufadienolides. Chemistry, biological activities and prospects for possibility of therapeutic use of *Kalanchoe* plant-derived bufadienolides have been presented. Some information on possible side effects of bufadienolides have been also included. The current review comprises data (to May, 2017) from journals recorded in international databases (Medline/Pubmed, Scopus, ScienceDirect/Elsevier, Springer Link/ICM) and other scientific journals, non-indexed in these databases.

## Bufadienolide structures and their concentration in *Kalanchoe* plants

**Fig. 1:**
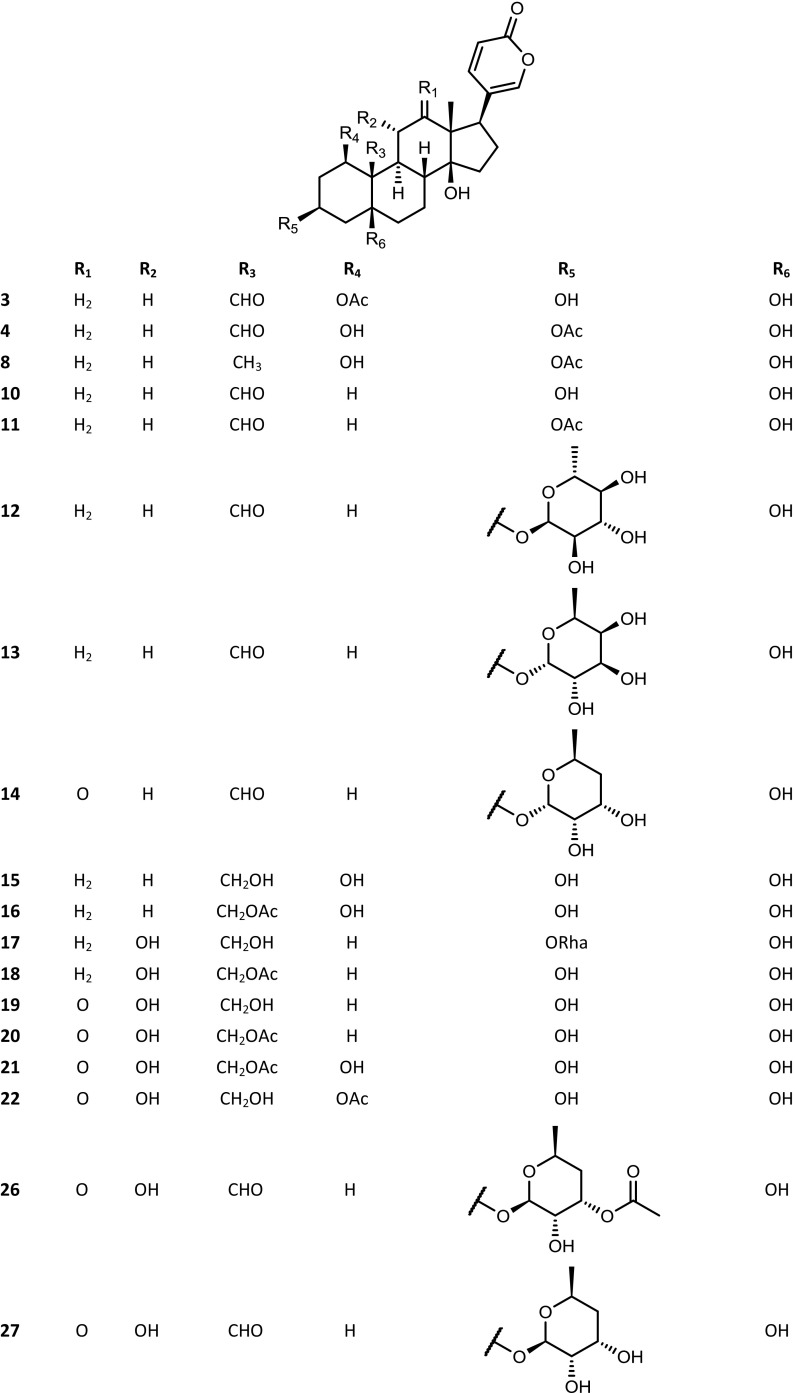

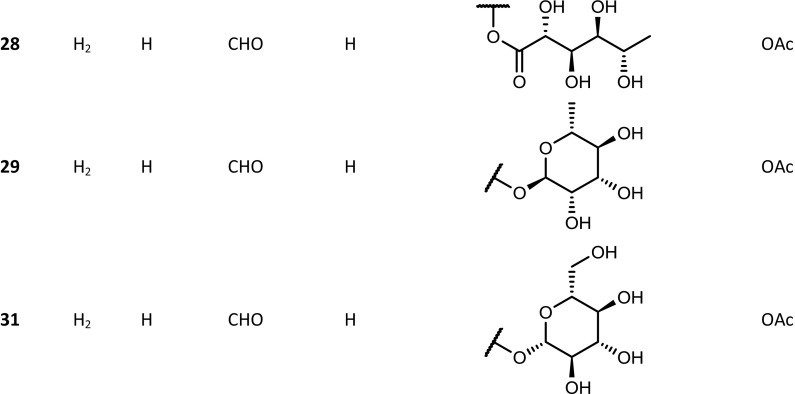
Structures of the compounds **3**, **4**, **8**, **10**–**22**, **26**–**29** and **31**

**Fig. 2:**
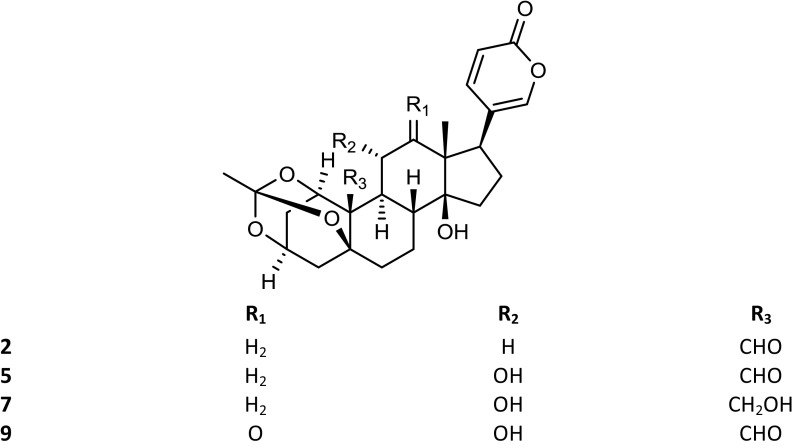
Structures of compounds **2**, **5**, **7** and **9**

**Fig. 3:**
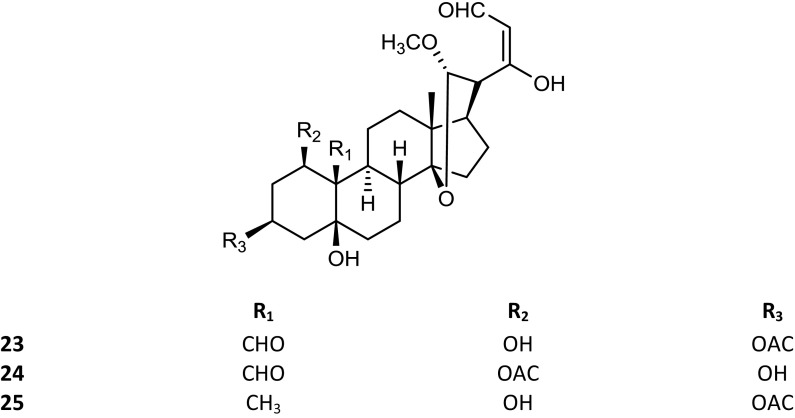
Structures of the compounds **23**, **24** and **25**

**Fig. 4:**
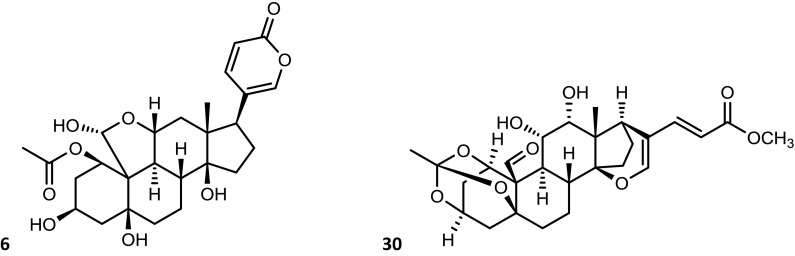
Structures of the compounds **6** and **30**

The *Kalanchoe* species are succulent plants. Their aerial parts were reported to contain not only steroid compounds, but also some flavonoids, phenolic acids, anthocyanins, alkaloids, saponins and tannins (El Abdellaoui et al. 2010; Chowdhury et al. 2011). The polyhydroxy C-24 structure of bufadienolides is based on a six-membered lactone (α-pyrone) ring, located at position C-17β. Some of these compounds have been isolated from *Kalanchoe* plants, and their structures have been established by spectral techniques. The available structures of these compounds are presented in the Figs. 1, 2, 3 and 4. Supratman et al. (2000) have identified two compounds including bryophyllin A (**5**) and bryophyllin C (**7**) from *K. pinnata*, and in 2001 five next compounds: bersaldegenin-3-acetate (**4**), bersaldegenin-1,3,5-orthoacetate (**2**), daigremontianin (**9**), bersaldegenin-1-acetate (**3**) and methyl daigremonate (**30**) in study on bufadienolides of *K. daigremontiana* × *tubiflora* (Fig. 1). Eight bufadienolides were identified by Wu et al. (2006), in the extract of aerial parts of *K. gracilis*, which included kalanchosides A (**12**), B (**13**) and C (**14**), thesiuside (**31**), hellebrigenin (**10**), hellebrigenin-3-acetate (**11**), bryophyllins A (**5**) and B (**6**). The systematic names of these compounds are shown in the Table 2. From roots of *K. daigremontiana*, eight new bufadienolides named as kalandaigremoside A (**15**), B (**16**), C (**17**), D (**18**), E (**19**), F (**20**), G (**21**) and H (**22**) were isolated and characterized by Moniuszko-Szajwaj et al. (2016).

**Table 2:** The systematic names of compounds 1–31

No.	Name	Systematic name	Species and plant organs	References
1		3β-(4′,6′-dideoxy-β-arabino-hexopyranosyloxy)-2β-acetoxy-5β,14β-dihydroxy-19-oxobufa-20,22-dienolide	*K. tomentosa* (leaves)	Rasoanaivo et al. (1993)
2	Bersaldegenin-1,3,5-orthoacetate = Melianthugenin	(1β,3β,5β)-1,3,5-[(1R)-ethylidynetris(oxy)]-14-hydroxy-19-oxobufa-20,22-dienolide	*K. daigremontiana* × *tubiflora* (leaves)	Supratman et al. (2001a, b)
3	Bersaldegenin-1-acetate	3-acetoxy-1,5,14-trihydroxy-19-oxobufa-20,22-dienolide	*K. daigremontiana* × *tubiflora* (leaves)	Supratman et al. (2001a, b)
4	Bersaldegenin-3-acetate	(1β,3β,5β)-3-(acetyloxy)-1,5,14-trihydroxy-19-oxobufa-20,22-dienolide	*K. daigremontiana* × *tubiflora* (leaves)	Supratman et al. (2001a, b)
5	Bryophyllin A = Bryotoxin C	[1β(R),3β,5β,11α]-1,3,5-ethylidynetris(oxy)-11,14-dihydroxy-19-oxo-bufa-20,22-dienolide	*K. pinnata* (leaves)	Supratman et al. (2000)
6	Bryophyllin B	(1β,3β,5β,8ξ,9ξ,10ξ,11α,19R)-1-acetoxy-3,5,14,19-tetrahydroxy-11,19-epoxybufa-20,22-dienolide	*K. gracilis* (aerial parts)	Wu al et. (2006)
7	Bryophyllin C	[1β(R),3β,5β,11α]-1,3,5-ethylidynetris (oxy)-11,14,19-trihydroxybufa-20,22-dienolide	*K. pinnata* (leaves)	Supratman et al. (2000)
8	Daigredorigenin-3-*O*-acetate	3-(acetyloxy)-1,5,14-trihydroxy-, (1β,3β,5β)-bufa-20,22-dienolide	*K. daigremontiana* (aerial parts and roots)	Wagner et al. (1985)
9	Daigremontianin	(1β,3β,5β,11α)-1,3,5-ethylidynetris(oxy)-11,14-dihydroxy-12,19-dioxobufa-20,22-dienolide	*K. daigremontiana* × *tubiflora* (leaves)	Supratman et al. (2001a, b)
10	Hellebrigenin	(3β,5β)-3,5,14-trihydroxy-19-oxobufa-20,22-dienolide	*K. gracilis* (aerial parts)	Wu et al. (2006)
11	Hellebrigenin-3-acetate	(3β,5β)-3-acetoxy-5,14-dihydroxy-19-oxobufa-20,22-dienolide	*K. gracilis* (aerial parts)	Wu et al. (2006)
12	Kalanchoside A	(3β,5β)-3-[(6-deoxy-α-d-glucopyranosyl)oxy]-5,14-dihydroxy-19-oxobufa-20,22-dienolide	*K. gracilis* (aerial parts)	Wu et al. (2006)
13	Kalanchoside B	(3β,5β)-3-[(6-deoxy-α-l-galactopyranosyl)oxy]-5,14-dihydroxy-19-oxobufa-20,22-dienolide	*K. gracilis* (aerial parts)	Wu et al. (2006)
14	Kalanchoside C	12-oxohellebrigenin-3-*O*-4,6-dideoxy-α-ribo-hexopyranoside	*K. gracilis* (aerial parts)	Wu et al. (2006)
15	Kalandaigremoside A	1β,3β,5β,14β,19-pentahydroxybufa-20,22-dienolide	*K. daigremontiana* (roots)	Moniuszko-Szajwaj et al. (2016)
16	Kalandaigremoside B	19-(acetyloxy)-1β,3β,5β,14-tetrahydroxybufa-20,22-dienolide	*K. daigremontiana* (roots)	Moniuszko-Szajwaj et al. (2016)
17	Kalandaigremoside C	3β-(*O*-α-l-rhamnopyranosyl)-5β,11α,14,19-tetrahydroxybufa-20,22-dienolide	*K. daigremontiana* (roots)	Moniuszko-Szajwaj et al. (2016)
18	Kalandaigremoside D	19-(acetyloxy)-3β,5β,11α,14-tetrahydroxybufa-20,22-dienolide	*K. daigremontiana* (roots)	Moniuszko-Szajwaj et al. (2016)
19	Kalandaigremoside E	3β,5β,11α,14β,19-pentahydroxy-12-oxo-bufa-20,22-dienolide	*K. daigremontiana* (roots)	Moniuszko-Szajwaj et al. (2016)
20	Kalandaigremoside F	19-(acetyloxy)-3β,5β,11α,14β-tetrahydroxy-12-oxo-bufa-20,22-dienolide	*K. daigremontiana* (roots)	Moniuszko-Szajwaj et al. (2016)
21	Kalandaigremoside G	19-(acetyloxy)-1β,3β,5β,11α,14β-pentahydroxy-12-oxo-bufa-20,22-dienolide	*K. daigremontiana* (roots)	Moniuszko-Szajwaj et al. (2016)
22	Kalandaigremoside H	1β-(acetyloxy)-3β,5β,11α,14β,19-pentahydroxy-12-oxo-bufa-20,22-dienolide	*K. daigremontiana* (roots)	Moniuszko-Szajwaj et al. (2016)
23	Kalanhybrin A	Chol-22-ene-19,24-dial, 3-(acetyloxy)-14,21-epoxy-1,5,22-trihydroxy-21-methoxy-, (1β,3β,5β,14β,21*S*,22*E*)-	*K. hybrida* (whole plant)	Kuo et al. (2008)
24	Kalanhybrin B	Chol-22-ene-19,24-dial, 1-(acetyloxy)-14,21-epoxy-3,5,22-trihydroxy-21-methoxy-, (1β,3β,5β,14β,21*S*,22*E*)-	*K. hybrida* (whole plant)	Kuo et al. (2008)
25	Kalanhybrin C	Chol-22-en-24-al, 3-(acetyloxy)-14,21-epoxy-1,5,22-trihydroxy-21-methoxy-, (1β,3β,5β,14β,21*S*,22*E*)-	*K. hybrida* (whole plant)	Kuo et al. (2008)
26	Kalantuboside A	Bufa-20,22-dienolide, 3-[(3-*O*-acetyl-4,6-dideoxy-α-l-*ribo*-hexopyranosyl)oxy]-5,11,14-trihydroxy-12,19-dioxo-, (3β,5β,11α)-	*K. tubiflora* (whole plant)	Huang et al. (2013)
27	Kalantuboside B	Bufa-20,22-dienolide, 3-[(4,6-dideoxy-α-l-*ribo*-hexopyranosyl)oxy]-5,11,14-trihydroxy-12,19-dioxo-, (3β,5β,11α)-	*K. tubiflora* (whole plant)	Huang et al. (2013)
28	Lanceotoxin A	[(3S,5S,8R,9S,10S,13R,14S,,17R)-5-acetyloxy-10-formyl-14-hydroxy-13-methyl-17-(6-oxopyran-3-yl)-2,3,4,6,7,8,9,11,12,15,16,17-dodecahydro-1H-cyclopenta[a]phenanthren-3-yl],(2R,3R,4S,5S)-2,3,4,5-tetrahydroxyhexanoate,C	*K. lanceolate* (whole plant)	Anderson et al. (1984)
29	Lanceotoxin B	[(3S,5S,8R,9S,10S,13R,14S,17R)-10-formyl-14-hydroxy-13-methyl-17-(6-oxopyran-3-yl)-3-[(2R,3R,4R,5R,6S)-3,4,5-trihydroxy-6-methyloxan-2-yl]oxy-2,3,4,6,7,8,9,11,12,15,16,17-dodecahydro-1H-cyclopenta[a]phenanthren-5-yl] acetate	*K. lanceolata* (whole plant)	Anderson et al. (1984)
30	Methyl daigremonate	Methyl[1β,3β,5β,11α,12α]-(22E)-1,3,5-ethylidynetris(oxy)-14,21-epoxy-11,12-dihydroxy-19-oxo-5β,14β-chola-20,22-dien-24-oate	*K. daigremontiana* × *tubiflora* (leaves)	Supratman et al. (2001a, b)
31	Thesiuside	5-*O*-acetylhellebrigenin 3-*O*-β-d-glucopyranoside	*K. gracilis* (aerial parts)	Wu et al. (2006)

The presence of daigredorigenin-3-*O*-acetate (**8**) in *K. daigremontiana* was reported as early as in the 80s of the twentieth century (Wagner et al. 1985). Next compounds were isolated in 2008 by Kuo et al. from the extract of *K. hybrid:* kalanhybrin A (**23**), B (**24**) and C (**25**). The only one new compound was isolated from *K. tomentosa* and it was 3β-(4′,6′-dideoxy-β-arabino-hexopyranosyloxy)-2β-acetoxy-5β,14β-dihydroxy-19-oxobufa-20,22-dienolide (**1**) (Rasoanaivo et al. 1993). Few years ago, two bufadienolide glycosides, i.e. kalantuboside A (**26**) and kalantuboside B (**27**) were found in the extract from the whole plant of *K. tubiflora* (Huang et al. 2013). Furthermore, it has been also established that *K. lanceolata* (Forssk.) Pers. synthesizes 5-*O*-acetylhellebrigenin glycosides, i.e. lanceotoxin A (5-*O*-acetylhellebrigenin 3-*O*-α-l-rhamnoate) (**28**) and lanceotoxin B (5-*O*-acetylhellebrigenin 3-*O*-α-l-rhamnopyranoside) (**29**) (Anderson et al. 1984). In the flower heads, leaves and stems extract of *K. tubiflora* and in the roots of the hybrid *K. tubiflora* × *pinnata*, three bryotoxin A, B and C were detected. In the extract from flower heads, leaves and stems of the hybrid, *K. daigremontiana* × *pinnata*, only bryotoxins B and C were found. No bryotoxins were detected in extract from *Kalanchoe fedtschenkoi* Raym.-Hamet & H. Perrier (McKenzie et al. 1987).

Quantification of bufadienolides in leaves of *K. pinnata* grown in Brazil and Germany (Oufir et al. 2015) revealed that bryophyllin A (**5**), bersaldegenin-3-acetate (**4**), bersaldegenin-1,3,5-orthoacetate (**2**) and bersaldegenin-1-acetate (**3**) are main bufadienolide components of these plant organs. In plants grown in Brazil, the total bufadienolide concentrations ranged from 16.28 to 40.50 mg/100 g of dry weight. The total content of bufadienolides in plant material from Germany was lower and attained from 3.78 to 12.49 mg/100 g of dry weight. Additional analyses of other species indicated that in leaves of *K. daigremontiana* and in stems of *K. tubiflora*, bersaldegenin-1,3,5-orthoacetate (**4**) was the predominant bufadienolide compound. Contrary to *K. pinnata*, the leaves of *K. tubiflora* contained very low amounts of bryophyllin A (**5**), bersaldegenin-3-acetate (**4**), bersaldegenin-1,3,5-orthoacetate (**2**) and bersaldegenin-1-acetate (**3**).

On the other hand, there are significant gaps in the available literature on the presence of bufadienolides in *Kalanchoe* species and their distribution in different organs of these plants. Our preliminary studies (unpublished data) suggested that the total content of bufadienolides varied in different plant parts. While bufadienolides content per gram of dried stems and roots *K. daigremontiana* was 65 and 395 µg, respectively, there was no occurrence of these compounds in the leaves. This distribution in the plant was quite unusual and probably reflected physiological and ecological function of these compounds. It is assumed that bufadienolides, similarly to other secondary metabolites, are involved in chemical plant protection against pathogenic microorganisms and herbivores. They are also recognized as precursors of hormonal substances and participate in the formation of membranous structures.

## Pharmacological actions of bufadienolides of various origins

The therapeutic effects of bufadienolide-containing preparations have been known from the ancient times. A bufadienolide-rich plant *Scilla maritima* was used by Egyptians to cure heart diseases. Bufadienolides are also the principal bioactive ingredient of a traditional Chinese drug Ch’an Su, containing the skin secretions of toads such as *Bufo gargarizans* Cantor and *Bufo melanostictus* Schneider. Currently, the most investigated pharmacological activities of bufadienolides of various origins are cardiotonic and anticancer properties. Other physiological actions of bufadienolides include blood pressure stimulating, antiangiogenic, antiviral, immunomodulatory and antibacterial activities (Gao et al. 2011; Kamboj et al. 2013; Wei et al. 2017). Biological activity of *Kalanchoe*-derived bufadienolides is a relatively new issue. For that reason, a number of reports directly related to molecular background of pharmacological action of bufadienolides isolated from *Kalanchoe* plants is limited. Contemporary literature mostly provides data on pharmacological actions and possible therapeutic significance of animal bufadienolides, however, some information on compounds that are also present *Kalanchoe* species is available.

The main molecular mechanism of pharmacological action of bufadienolides and their derivatives involves the induction of a local increase of Na^+^ as a result of inhibition of a carrier enzyme: Na^+^/K^+^-ATPase (EC 3.6.1.37; the sodium pump), commonly described as a “digitalis-like” effect. Na^+^/K^+^-ATPase is responsible for maintaining of electrochemical gradient of Na^+^ and K^+^ through the cell membrane. The keeping of low Na^+^ and high K^+^ intracellular concentrations and membrane potential is critical for excitability of nerves and muscle cells (including cardiomyocytes) as well as for the secondary active transport. Bufadienolides have the ability to alter myocardial ion balance resulting in an increase of intracellular Ca^2+^ concentration ([Ca^2+^]i) via a backward-running of Na^+^/Ca^2+^ exchanger, and as a consequence, leading to contractions of cardiac and arterial myocytes (Melero et al. 2000; Schoner and Scheiner-Bobis 2007). Additionally, studies on numerous cell lines confirmed the anticancer properties of different bufadienolides (Kamboj et al. 2013) and provided some information on anticancer mechanisms and selective toxicity of bufadienolides towards malignant cells. Studies on human liver microsomes (HLMs) indicated that hydroxylation and dehydrogenation might be the major metabolic pathways of bufadienolides (Han et al. 2016). Molecular mechanisms of anticancer activities of hellebrin and its aglycone hellebrigenin (compounds that were also found in *Kalanchoe* plants) were described by Moreno et al. (2013). According to those authors, both compounds are able to bind to the alpha subunits of the Na^+^/K^+^-ATPase and display similar growth inhibitory effects in different cancer cell lines, i.e. A549 (lung cancer), U373 (glioblastoma astrocytoma), Hs683 (glioma), T98G (glioblastoma), MCF-7 (breast adenocarcionoma), SKMEL-28 (melanoma), PC-3 (prostate cancer) and HT-29 (colorectal cancer). For hellebrin, the growth inhibitory concentrations at 50% (IC_50_) were estimated as 6–58 nM, while the IC_50_ for hellebrigenin ranged from 3 to 42 nM. Other experiments (Yuan et al. 2016) conducted on human glioblastoma U-87 cell line and a pancreatic SW1990 cancer cell line demonstrated that gamabufotalin and arenobufagin (bufadienolides of animal origin) possessed selective cytotoxic activity against tumour cells rather than normal cells (peripheral blood mononuclear cells, PBMCs). Both bufadienolides (at the final concentrations of 1.6, 8, 40, 200 and 1000 ng/ml) displayed dose-dependent anticancer effects, when compared to control (untreated) cells. For gamabufotalin, IC_50_ values were 16.8 ± 6.5 and 8.1 ± 1.5 ng/ml in the U-87 and SW1990 cells, respectively. Arenobufagin action was characterized by IC_50_ 10.3 ± 3.3 and 9.9 ± 2.2 ng/ml, in the U-87 and SW1990 cells, respectively. Moreover, the authors suggested that gamabufotalin might be a promising candidate for using as an adjuvant therapeutic agent. This opinion was based on data originated from experiments on PBMCs treated with bufadienolides at nontoxic concentrations, which resulted in a modulation of fractions of CD4 + CD25 + Foxp3 + regulator T (Treg) cells in mitogen-activated PBMCs. In pathophysiology of cancer and haematologic malignancies, Treg cells were found to play a critical role in development of tumour immunotolerance by suppressing the host response to tumour immunity. Thus, by decreasing the amount and activity of these cells, gamabufotalin may enhance the efficiency of conventional anticancer drugs (Yuan et al. 2016). Studies of Zhang et al. (2016) revealed that the treatment of A549 cell line with gamabufotalin (5–500 nM) significantly reduced viability of the cells, when compared to the control (untreated A549 cells). For the 48 h-treatment, the IC_50_ value was 48.4 ± 2.5 nM. Moreover, no cytotoxicity was found in analogous experiments on human normal lung cell line (HLF cells). Molecular mechanisms of cytotoxic action of the bufadienolide involved the G2/M cell cycle arrest and induction of apoptosis in A549 cells. In vivo, gamabufotalin (10 or 20 mg/kg of body weight) was able to down-regulate the protein level of Hsp90 in tumor tissues of the xenograft mice, when compared to control animals (treated with phosphate-buffered saline) (Zhang et al., 2016). Furthermore, in studies of other scientists (Yu et al. 2014), gamabufotalin (10, 50 and 100 nM) suppressed the expression of cyclooxygenase 2 (COX-2) in lung cancer cells, in comparison to the dimethyl sulfoxide (DMSO) vehicle control group. Biochemical mechanisms of this anti-inflammatory action of gamabufotalin involve the inhibition of the phosphorylation of inhibitor of nuclear factor kappa-B (IκB), which prevents the translocation of nuclear factor kappa B (NF-κB) to nucleus and, in consequence, halts the recruitment of NF-κB and p300 on COX-2 promoter (Yu et al. 2014). As another potential mechanism of antitumour activity of gamabufotalin, the inhibition of the vascular endothelial growth factor (VEGF)-induced angiogenesis by suppressing vascular endothelial growth factor receptor 2 (VEGFR-2) signaling pathway has been also suggested (Tang et al. 2016).

Recently published data (Bachmann et al. 2017) suggest that the bufadienolide-enriched fraction from *K. pinnata* leaf juice (containing bersaldegenin-1-acetate, bryophyllin A, bersaldegenin-3-acetate, bersaldegenin-1,3,5-orthoacetate as well as two unidentified compounds: flavonoid (*m/z* 581, [M + H]^+^, 303 (aglycone)) and bufadienolide *m/z* 477 ([M + H]^+^) display biological activity that might be useful in the treatment of overactive bladder. The examined fraction (0.01–1 mg/ml) had the inhibitory effect on detrusor contractility in vitro. The inhibition was dose-dependent, and no such effects were found for flavonoid fraction isolated from the leaf juice.

## Biological activity of *Kalanchoe* species-derived bufadienolides

### Anticancer effects

The existing evidence of anticancer properties of bufadienolides originates mostly from research on compounds isolated from animal sources, particularly of toad venom (Takai et al. 2012). However, reports indicating on the chemopreventive effects of *Kalanchoe* bufadienlides are also available. Bryophyllin B, isolated from *Bryophyllum pinnatum* (Lam.) Oken (*K. pinnata*) was shown to be a potent cytotoxic agent against the KB cell line, with the ED_50_ value <80 ng/ml (Yamagishi et al. 1989). Studies on 8 bufadienolides, including kalanchosides A–C, isolated from the aerial parts of *K. gracilis* Hance revealed considerable cytotoxic/anticancer activities of all isolated compounds against several human tumour cell lines such as nasopharyngeal (KB) and its MDR variant (KB-VIN), lung (A549), ovarian (1A9), prostate (PC-3), ileocecal (HCT-8), and epidermoid (A431) cells. Mostly, effectiveness of the examined bufadienolides was higher than the effect of etoposide (a reference cytostatic/anticancer drug) and attained the nanomolar range of their concentrations (Wu et al. 2006). Furthermore, bryophyllin B was able to inhibit the replication of HIV in H9 lymphocytes, at the ED_50_ value of <0.25 µg/ml and therapeutic index of >6.27 µg/ml. Additionally, Huang et al. (2013) demonstrated that bufadienolide glycosides isolated from *K. tubiflora* displayed strong cytotoxicity against four human cancer cell lines: A549, Cal-27 (oral adenosquamous carcinoma), A2058 (melanoma) and HL-60 (promyelocytic leukemia). Bufadienolide effects were assessed in comparison with positive controls, i.e. mitomycin-C and cycloheximide, while 0.05% DMSO-treated samples were used as vehicle controls. IC_50_ values for the examined glycosides ranged from 0.01 to 10.66 µM. For mitomycin-C, IC_50_ ranged from 4.63 to 9.34 µM, while IC_50_ for cycloheximide was detectable only in experiments on HL-60 cells and attained 40.60 µM (Huang et al. 2013). The cytotoxic effect against tumour cell lines was also found in experiments with bufadienolides isolated from the crude methanol extract of *K. hybrida* Desf. ex Steud. Anticancer activity of the isolated compounds (4 and 20 µg/ml) was evaluated in experimental models of three cancer cell lines, i.e. MCF-7, NCI-H460 (large cell lung cancer), and SF-268 (anaplastic astrocytoma), using actinomycin D (10 mM) and DMSO (0.3%) as positive and vehicle controls, respectively. The strongest cytotoxic effects (even up to 100% of growth inhibition) towards the examined cells were found for bersaldegenin 3-acetate and daigredorigenin 3-acetate (Kuo et al. 2008).

### Cardiotonic effects

The cardiac glycoside-like effects of a bufadienolide compound, extracted from *K. daigremontiana,* were demonstrated by Scholtysik et al. (1986). During the studies on animals, the authors observed pharmacological effects similar to those evoked by digitalis glycosides. The IC_50_ for Na^+^/K^+^-ATPase activity in vitro was estimated as 1.4 × 10^−7^ M, while for ouabain (a reference compound) this parameter was 2 × 10^−7^ M. Intravenous infusion of the examined bufadienolide to guinea-pigs with a rate of 20 µg/kg/min resulted in ventricular arrhythmias and death after accumulated doses of about 760 and 860 µg/kg of body weight, respectively. However, in general, the examined bufadienolide was less toxic than ouabain.

### Anti-viral activity

Bufadienolides isolated from leaves of *K. pinnata* and *K. daigremontiana* × *tubiflora* are able to inhibit the activation of Epstein-Barr virus early antigen (EBV-EA) in Raji cells, induced by 12-*O*-tetradecanoylphorbol-13-acetate. Bryophyllin A had the strongest inhibitory effect (IC_50_ = 0.4 µM), while compounds lacking the orthoacetate moiety such as bryophyllin C and bersaldegenin-3-acetate possessed significantly lower activities (IC_50_ = 1.6 and 3 µM, respectively), when compared to control samples (untreated with the bufadienolide) (Supratman et al. 2001a, b).

### Inhibition of serine proteinases

So far, research on bufadienolides and enzyme interactions has been focused only on the inhibition of ATP-ase activity. The issue of inhibition of other groups of enzymatic proteins by bufadienolides has appeared in the literature within last 2 years. In 2015, Shibao and co-authors published results from studies on a serine proteinase inhibitor, isolated from *Rhinella schneideri* (Schneider’s toad) poison. The inhibitor was identified as lithocholic acid, a biosynthetic precursor of bufadienolide. In spite of the fact that the study was conducted on animal-derived preparation, it should be mentioned as the first report confirming that bufadienolide-type compound might suppress the enzymatic activity of serine proteinase. Thus, the influence of *Kalanchoe*-derived bufadienolides on enzymatic properties of serine proteinases still is very poorly evidenced. Inhibitory action of bufadienolide-rich fraction from *K. daigremontiana* on enzymatic activity of thrombin has been recently described by Kolodziejczyk-Czepas et al. (2017). Native (untreated with the examined fraction) thrombin was used as a control sample. A serine proteinase enzyme—thrombin (plasma coagulation factor II), is responsible for the formation of fibrin clot, and thus, for the prevention of uncontrolled blood loss after injury of blood vessel. In the above in vitro study, bufadienolide-rich fraction inhibited enzymatic activity of thrombin with IC_50_ = 2.79 µg/ml. The efficacy of a reference compound (direct inhibitor of thrombin)—argatroban (anti-thrombotic drug) was characterized by IC_50_ = 0.78 µg/ml. On the other hand, analysis of kinetic parameters of the reaction indicated that *K. daigremontiana* fraction contains compounds with diverse inhibitory mechanisms, when compared to argatroban. Components of the investigated fractions were uncompetitive inhibitors of thrombin. *In silico* studies on interactions of the most common compounds, identified in the examined bufadienolide-rich fraction to crystal structure of thrombin were also conducted. The obtained results indicated that for the inhibitory effect of *K. daigremontiana* fraction, most likely the presence of compounds such as bersaldegenin-1,3,5-orthoacetate, bersaldegenin-1-acetate, bersaldegenin, hovetrichoside C, deigredorigenin-3-acetate is responsible.

### Bufadienolides as antioxidants?

Due to hydrophobic, steroid structure of bufadienolide-type compounds, antioxidant properties of those substances are considered to be weak. However, existing evidence indicated that bufadienolides possess some antioxidant potential. Moreover, this group of compounds may be a base for development of new derivatives with enhanced antioxidant properties and decreased toxicity (obtained by chemical alterations of the pyrone moiety) (Aucamp 2014). Recent studies on bufadienolide-rich fraction of *Kalanchoe daigremontiana* roots demonstrated that its DPPH^·^ scavenging ability was characterized by EC_50_ = 21.80 μg/ml (Kolodziejczyk-Czepas et al. 2016). Under the same experimental conditions, for the reference compounds, i.e. Trolox and (−)-epicatechin, EC_50_ values were 4.64 and 3.30 μg/ml, respectively. It should be emphasized that earlier results, obtained by other authors in analogous experiments on different *Kalanchoe* plants indicated on significantly lower antioxidant efficacy of extracts, originated from different organs of *Kalanchoe* species (Sharker et al. 2012; Quazi Majaz et al. 2011a, b). Furthermore, antioxidant action of the mentioned bufadienolide-rich extract of *Kalanchoe daigremontiana* was also confirmed using an experimental model of blood plasma exposed to peroxynitrite-induced oxidative stress (Kolodziejczyk-Czepas et al. 2016).

### Other biological actions of *Kalanchoe* bufadienolides

Toxic (insecticidal) action of daigremontianin and bersaldegenin-1,3,5-orthoacetate, isolated from the leaves of *K. daigremontiana*, was demonstrated using an experimental system of larvae of *Bombyx mori* (Maharani et al. 2008).

## The risk of side effects and prospects for pharmacological use of bufadienolides occurring in *Kalanchoe* species

During the last 10 years, a growing interest in the evaluation of the metabolome of *Kalanchoe* plants and biological activities of *Kalanchoe*-derived extracts and substances, including bufadienolides has been observed. For instance, from the total amount of 483 publications containing the name “*Kalanchoe*” available in the Medline/Pubmed database, over 150 records derive from the last 10 years (data from 30 March, 2017; search criteria “*Kalanchoe*”). On the other hand, contrary to in vivo studies, confirming pharmacological effects of composed extracts isolated from different *Kalanchoe* species, physiological effects of bufadienolides extracted from these plants and their safety have been poorly described. The vast majority of reports on pharmacological activity of different *Kalanchoe*-based drugs still derive from traditional medicine and concern preparations based on crude extracts. However, studies on standardized preparations from *Kalanchoe* species also are available. According to data from a service of the U.S. National Institutes of Health “ClinicalTrials.gov” (https://clinicaltrials.gov/ct2/home, data from 30 March, 2017; search criteria “*Kalanchoe*” or “*Bryophyllum*”), four clinical studies on *Bryophyllum pinnatum*/*K. pinnata* have been recorded. Furthermore, after using a word “bufadienolide” four another results have been appeared, while a combination of “*Kalanchoe*” and “*bufadienolide*” have not provided any results. No information on animal or clinical studies on therapeutic effects of bufadienolides isolated from *Kalanchoe* was found in Medline/Pubmed, Scopus, ScienceDirect/Elsevier and Springer Link/ICM databases (data from 30 March, 2017). Biological activities of bufadienolides that are synthetized by these plants are very promising from a pharmacological point of view, however, they have been mostly studied in vitro. Therefore, nowadays, only a preliminary indication of the most promising prospects for pharmaceutical uses of bufadienolides is possible.

The therapeutic use of most bufadienolides is limited by to a narrow therapeutic index and risk of development of cardiotoxicity (Cheng 2001; Pamnani et al. 1994). A risk of toxicity of bufadienolide-containing plant extracts is inadequately evaluated. For example, no toxicity of the bufadienolide-rich *K. daigremontiana* fraction on blood platelets was found in vitro (Kolodziejczyk-Czepas et al. 2016). On the other hand, studies in South Africa indicated that ingestion of cumulative neurotoxic various plant-derived bufadienolides such as cotyledoside, tyledosides, orbicusides and lanceotoxins is a potential risk to humans (Botha 2016). Hence, numerous investigations have been developed to generate chemical and biotransformed bufadienolide derivatives or analogues with effective therapeutic action and considerably reduced toxicity. The in vitro biotransformations of natural bufadienolides have been conducted in various systems—in plant cell suspension cultures, fungi and bacteria (Gao et al. 2011). Some of these modified bufadienolides were able to selectively kill malignant cells. Studies of Daniel et al. (2003) showed this preferential cytotoxic action towards malignant cells for both a natural cardioactive bufadienolide—hellebrin (0.1–100 µM) as well as for its three derivatives (100 µM), lacked the cardioactive properties. Medium for the controls was supplemented with corresponding amounts of the used bufadienolide vehicle. While normal peripheral blood mononuclear cells were affected to a minimal extent, the examined substances induced the caspase-dependent pathway and initiated apoptosis in Jurkat T lymphoblasts. Since the therapeutic use of bufadienolides in anti-cancer therapy is limited by their influence on heart physiology, a considerable potential of using in cancer therapy may have compounds possessing a tumour-specific cytotoxicity with simultaneous lack of cardiac activity (Daniel et al. 2003). Current research of bufadienolides (hellebrigenin, among others) also covers some pharmacokinetic aspects of their interactions with human serum albumin (HSA), the main carrier of various drugs. *In vitro* and in silico analyses indicated that the binding affinity for HSA of various bufadienolides is considerably related to differences in their structures. The presence of C=O bond at the C12 position decreased the binding affinity for HSA, while other polar groups increased the bufadienolide affinity to HSA. In particular, the presence of 11-OH or 16-OAc groups may be important for anchoring bufadienolides within site I of the HSA pocket. The 11-OH or 16-OAc-mediated interactions of bufadienolide and HSA involve the hydrogen bonding (H-bonding) with protein Tyr150 or Lys199 groups, respectively (Zhou et al. 2015).

Another way of the enhancing the therapeutic effect and reducing the toxicity of anticancer drugs such as bufadienolides may be preparation of long-circulating, poloxamer-modified liposomes. According to Hu et al. (2011), these liposomes have significantly prolonged retention time, when compared to bufadienolide solutions and unmodified liposomes. The LD_50_ value of modified liposomes was about 3.5 times higher than the LD_50_ recorded for bufadienolide solution (i.e. 4.48 and 1.28 mg/kg, respectively). The use of bufadienolide liposomes resulted in a considerable increase of anti-tumour efficiency both in mice bearing H22 liver cancer cells and Lewis pulmonary cancer cells (2.15 and 2.96 times, respectively), compared to the anticancer effects observed in animals treated with bufadienolide solution. Promising results have been obtained by Mexican scientists (Alvarado-Palacios et al. 2015) in experiments on using nanocapsules containing the aquoethanolic extract from *K. daigremontiana* as selective anticancer agents. The nanocapsuled extract was characterized by higher cytotoxic efficacy (IC_50_ = 48.53 μg/ml) towards MDA-MB-231 metastatic breast cancer cell line, when compared to the non-encapsulated aquoethanolic extract (IC_50_ = 61.29 μg/ml). Moreover, studies on non-cancerous breast cell line MCF 10A revealed no cytotoxic effect of the nanocapsules containing the aquoethanolic extract of *K. daigremontiana* (at concentrations ≤200 μg/ml), whereas the non-encapsulated extract displayed significant cytotoxic effect (IC_50_ = 100.2 μg/ml).

## Conclusions

Ethnomedicinal plants of the *Kalanchoe* genus may be regarded as a new source of bufadienolides, since synthesis of these substances has been confirmed for these species. At present, however, toad venom remains the main source of these compounds. On the other hand, a growing number of reports have confirmed that *Kalanchoe*-derived bufadienolides display a wide range of biological actions, including cardiotonic, anticancer, anti-viral and other properties. Despite these promising findings, the therapeutic use of *Kalanchoe* plants is considerably limited by the lack of clinical evidence. Therefore, further studies on medicinal applications of bufadienolides and extracts of *Kalanchoe* species origin are required.
